# Correction: Developing a comprehensive inventory to define harm reduction housing

**DOI:** 10.1186/s12954-025-01290-0

**Published:** 2025-08-18

**Authors:** Sofia Zaragoza, Joseph Silcox, Sabrina Rapisarda, Charlie Summers, Patricia Case, Clara To, Avik Chatterjee, Alexander Y. Walley, Miriam Komaromy, Traci C. Green

**Affiliations:** 1 Heller School for Social Policy & Management, Opioid Policy Research Collaborative, Brandeis University, Waltham, MA USA; 2 Department of Sociology, University of MA–Boston, Boston, MA USA; 3 School of Criminology and Justice Studies, University of MA–Lowell, Lowell, MA USA; 4 Northeastern University, Bouve College of Health Sciences, Boston, MA USA; 5 Grayken Center for Addiction and Clinical Addiction Research and Education (CARE) Unit, Section of General Internal Medicine, Boston Medical Center, Boston, MA USA; 6 Boston University, Boston University Chobanian & Avedisian School of Medicine, Boston, MA USA; 7 Departments of Emergency Medicine and Epidemiology, Brown University Schools of Medicine and Public Health, Providence, RI USA

**Correction to: Harm Reduction Journal (2025) 22:11** 10.1186/s12954-025-01156-5

The following section headings: "Front desk security stations", "Common area", "Lockers" and "Designated smoking areas" in this article [1] are also mentioned in the legends of figures 1-4.

The section headings are removed from the article.

The original article has been corrected.
