# Supplementary material for: Developing a comprehensive inventory to define harm reduction housing
Source: Harm Reduct J. 2025 Jan 23;22:11. doi: 10.1186/s12954-025-01156-5 (PMC11756173; doi:10.1186/s12954-025-01156-5)
Supplement: Supplementary file 1 — Supplementary Material 1 [file 12954_2025_1156_MOESM1_ESM.docx]

**Supplementary Table 1: Harm Reduction Housing Inventory**

*Version 1.0: 32-items, Interviewer-administered, Resident version*

Instructions:

Preface inventory administration with a summary of its content: “I'd like to learn more about the location you are staying. I'm going to ask you about some services, supplies and policies there. Please tell me if you are aware of its availability, how frequently you use it, and how helpful it is for you to stay safe, including from overdose and other drug-related harm.”

For each inventory item do the following steps:

Step 1: Ask resident if they are aware of the service, marking zero if unaware and one if aware.

Step 2: Ask resident how frequently they use this service on a scale of 1 to 5, 1 being no use and 5 being used very frequently, recording the value they provide.

Step 3: Ask how helpful they feel the item is for keeping them safe including from drug related harm from a scale of 1-5 where 1 is not at all helpful and 5 is extremely helpful.

Step 4: For items where frequency is not applicable, the frequency section will be blocked off, only prompting you to ask the resident about their awareness and perceived helpfulness of the item.

| Are you aware of the availability of the following services, supplies, or policies at this location (the HRH site you are staying)? | Aware of availability | If aware, ask: How frequently do you use this? | If frequency is >0, ask:  How helpful is this for staying safe, including from overdose  and other drug-related harm? |
| --- | --- | --- | --- |
|  | (1=aware, 0=not aware) | (Response scale 1 to 5: Where 1= no use and 5=use very frequently) | (Response scale 1 to 5:  Where 1=not at all helpful and 5=extremely helpful) |
| Access to medication for substance use disorder on site or nearby (buprenorphine, methadone, naltrexone, Antabuse) |  |  |  |
| Access to HIV testing on site |  |  |  |
| Access to HIV medication for prevention and treatment (e.g., PrEP, antiretrovirals) |  |  |  |
| Medications are delivered to residents directly on site |  |  |  |
| A nurse or medical staff on site 24/7 |  |  |  |
| Access to wound care supplies or staff on site to help with wounds |  |  |  |
| Access to mental health care on site (e.g., counselor, therapist) |  |  |  |
| Availability of harm reduction supplies 24/7 (e.g., kits) |  |  |  |
| Access to sterile syringes |  |  |  |
| Access to pipes and other materials for safer smoking |  |  |  |
| Availability of Narcan kits |  |  |  |
| Area/space dedicated to substance use consumption on or near the site |  |  |  |
| Smoking area or outdoor space for smoking on site |  |  |  |
| A common area or community room on site |  |  |  |
| Lockers on site for resident use |  |  |  |
| Housekeeping of individual rooms by a cleaning crew |  |  |  |
| Ability to have an outside visitor in your room |  |  |  |
| Daily meals are provided/offered |  |  |  |
| Connected to a peer recovery coach on site |  |  |  |
| Connected to a case manager on site |  |  |  |
| Events or activities held to support those in recovery / those who wish to get started with recovery, such as support groups on site |  |  |  |
| Scheduled community meetings for staff and residents |  |  |  |
| Provides public transportation support (vouchers, ride share options) |  |  |  |
| Staff has Narcan on person at all times |  | X |  |
| Metal detectors before entry to site |  | X |  |
| Security guards on site |  | X |  |
| Consistent and clear rules or policies related to behavioral misconduct, such as behavioral warnings |  | X |  |
| Women centered and gender aware services, supplies, policies (e.g., pregnancy testing, contraceptives, hormone replacement therapy) |  | X |  |
| Absence policy on site (are you able to leave overnight or for extended periods without losing your space?) |  | X |  |
| Opportunities for paid or unpaid jobs or other activities that residents can be involved with |  | X |  |
| Room checks / wellness checks conducted by staff |  | X |  |
| Substance use tolerated in rooms (can you use in your room?) |  | X |  |

Scoring instructions:

Calculate an *Awareness* score at the item level by counting responses indicated as 1. For a scale level awareness score, sum the number of items indicated as 1 (aware).

For a *Frequency* score at the item level, count responses of frequencies across the range of response options. A frequency of use proportion can be calculated at the item-level and overall by summing responses of frequencies greater than none (i.e., at least some use) divided by the number of respondents who are aware of the component.

For a *Helpfulness* score, responses may be summed at the item and overall level, reported as median, mean, or mode depending on the distribution of responses.
