# Supplementary material for: Developing a comprehensive inventory to define harm reduction housing
Source: Harm Reduct J. 2025 Jan 23;22:11. doi: 10.1186/s12954-025-01156-5 (PMC11756173; doi:10.1186/s12954-025-01156-5)
Supplement: Supplementary file 2 — Supplementary Material 2 [file 12954_2025_1156_MOESM2_ESM.docx]

**Supplementary Table 2: Harm Reduction Housing Site Specific Components**

| **Inventory Items** | **Site 1** | **Site 2** | **Site 3** | **Site 4** | **Site 5** | **Site 6** |
| --- | --- | --- | --- | --- | --- | --- |
| **MOUD on Site:** Provides medications for opioid use disorder on site or in a nearby location | Yes | No | Yes | Yes | Yes | Yes |
| **HIV Testing:** Provides HIV testing as an onsite service | Yes | No | Yes | No | No | Yes |
| **HIV Prevention:** Provides HIV prevention medication on site (e.g., PrEP) | Yes | Yes | No | No | No | Yes |
| **HIV Treatment:** Provides medications (e.g., ART) used to treat HIV on site | Yes | Yes | No | No | No | Yes |
| **Security:** Security guards present on site | Yes | Yes | Yes | Yes | Yes | Yes |
| **HR supplies/ Syringes** | Yes | Yes | Yes | Yes | Yes | No |
| **HR Supplies/ Safe smoking supplies** | Yes | Yes | Yes | Yes | Yes | No |
| **HR Supplies/ Narcan** | Yes | Yes | Yes | Yes | Yes | Yes |
| **HR Services/ Nurse 24/7:** Nurse or medical staff present at all times on site | Yes | No | Yes | No | No | Yes |
| **HR Services/ Room Checks:** Room checks conducted as part of overdose prevention protocol | Yes | Yes | Yes | Yes | Yes | Yes |
| **HR Services/ Wound Care:** Wound care supplies and services provided on site | Yes | Yes | Yes | Yes | Yes | Yes |
| **HR Services/ Mental Health Care:** Behavioral health professional available to residents on site | No | Yes | No | No | No | Yes |
| **HR Supplies Availability:** Harm reduction supplies/kits available to residents 24/7 | Yes | No | Yes | Yes | Yes | No |
| **Staff Narcan:** Staff carry Narcan on person at all times | Yes | Yes | Yes | Yes | Yes | Yes |
| **Peer Recovery Coach:** Recovery coach on site to assist residents | Yes | No | Yes | No | No | No |
| **Metal Detectors:** Onsite required before entry into HRH space | Yes | No | No | Yes | Yes | Yes |
| **Smoking Area or Outdoor Space** | Yes | Yes | Yes | Yes | Yes | Yes |
| **Community Room/Common space:** Common area where residents can spend time | No | Yes | Yes | Yes | Yes | Yes |
| **Recovery Activities:** Events or activities held to support those in recovery who wish to get started with recovery, such as support groups | No | No | No | Yes | Yes | No |
| **Behavioral Policy:** Has rules or policies related to behavioral misconduct, such as behavioral warnings | Yes | Yes | Yes | Yes | Yes | Yes |
| **Housekeeping in Individual Rooms:** Frequent housekeeping of individual rooms by a cleaning crew | No | Yes | Yes | No | No | No |
| **Cleaning Crew/ Housekeeping:** Outside cleaning crew comes to clean common spaces | Yes | Yes | Yes | Yes | Yes | No |
| **Community Meetings:** Frequent scheduled meetings attended by staff and residents | Yes | Yes | Yes | Yes | Yes | No |
| **Intake Process:** When residents first arrive, staff collects information on resident and explains  policies and resources available to them | Yes | Yes | Yes | Yes | Yes | Yes |
| **Offboarding Process:** Provides information or resources to help residents with the transition to permanent housing | Yes | Yes | Yes | Yes | Yes | Yes |
| **Cameras:** Security cameras located throughout the site, inside and outside | Yes | Yes | Yes | Yes | No | Yes |
| **Lockers:** Lockers on site for residents to use | Yes | No | Yes | Yes | Yes | Yes |
| **Tolerance on Site:** Staff tolerant of substance use on site outdoors (tolerance defined as there are minimal to no consequences for use on site) | Yes | Yes | Yes | Yes | Yes | No |
| **Tolerance in Room:** Staff tolerant of substance use in rooms | Yes | Yes | Yes | No | No | No |
| **Transportation support:** Site provides public transportation support to residents | No | No | Yes | Yes | Yes | No |
| **Access to public transportation:** Public transportation reasonable walking distance from the site | Yes | Yes | Yes | Yes | Yes | Yes |
| **Flexibility:** Flexible policies/exceptions for certain people (e.g., holding a bed for someone who goes on vacation) | Yes | Yes | Yes | Yes | Yes | Yes |
| **Dedicated Consumption Space:**  Dedicated space for smoking or consuming substances | No | No | Yes | Yes | No | Yes |
| **Medication delivery:** Direct delivery of medications to residents on site | Yes | Yes | Yes | Yes | No | Yes |
| **Peer-to-peer security:** Residents involved in maintaining security - reporting activities, keeping watch, etc. | No | No | No | No | No | No |
| **Staff Harm Reduction Training:** Staff trainings on harm reduction practices and ideologies to implement in their practice at work | Yes | Yes | Yes | Yes | Yes | Yes |
| **Case Managers:** Residents can connect with case managers on site | Yes | Yes | No | Yes | Yes | Yes |
| **Meals**: Daily meals are provided/offered to residents | Yes | Yes | Yes | Yes | Yes | Yes |
| **Pro-social policies/capacities:** Able to room/live with a friend | Yes | Yes | Yes | No | Yes | No |
| **Pro-couple policies/capacities:** Able to room/live with partner | Yes | Yes | Yes | No | No | No |
| **Absence**: Length of time residents can be absent prior to losing bed/room without providing a reason or staff not made aware of location | 7 days | 7 days | 7 days | 7 days | 7 days | 5 days |
| **Gender affirming care services:** Site connects or provides gender aware services such as contraceptives, hormone replacement therapy, etc. | Yes | No | No | No | Yes | Yes |
| **Note: This table was compiled between June–September 2023. Policies and supplies are subject to change in HRH and thus items in this inventory could have changed since populated.* | | | | | | |
