# Supplementary material for: Developing a comprehensive inventory to define harm reduction housing
Source: Harm Reduct J. 2025 Jan 23;22:11. doi: 10.1186/s12954-025-01156-5 (PMC11756173; doi:10.1186/s12954-025-01156-5)
Supplement: Supplementary file 3 — Supplementary Material 3 [file 12954_2025_1156_MOESM3_ESM.docx]

**Supplemental Material 3: Key Informant Interview Guide**

1. Tell me about your position and role at the HRH location.
2. How would define harm reduction?
   1. What does harm reduction look at your site?
3. What are your perceptions about the most recent (11/23) removal and relocation of the individuals living in the Mass & Cass area?
   1. What are the effects of recent street clearings?
   2. What are the effects of influx of immigrants?
4. Tell me about your program at your HRH location?
   1. What harm reduction resources and supplies are available to residents?
   2. How are residents accessing harm reduction materials?
   3. Do residents have 24-hour accessibility to harm reduction materials?
   4. Do residents have access to HIV testing and treatment?
      1. Probe about PrEP, ARTs, etc.
5. Tell me about staffing at your site
   1. What type of harm reduction training do staff undergo?
   2. How does staff turnover effect site operations and community?
6. What is your sense of syringe disposal options and practices at your HRH location?
   1. How do residents dispose of used syringes?
   2. Are there any barriers to disposal?
7. Tell me about your overdose prevention protocol (e.g. Room checks) on site
   1. How are residents staying safe while using?
   2. What are the barriers or challenges associated with overdose prevention strategies?
8. How is smoking (of drugs) viewed at your site?
   1. Are there designated spaces or permissions to smoke in the rooms?
   2. Are materials for safer smoking available onsite or through a community partner?
9. Tell me about the security at your HRH location?
   1. Are peers involved in peer-to-peer security?
   2. Do you have policies relating to amnesty guests? Are non-residents allowed to sleep on site?
10. What is your understanding of the housing placement process (placement to your site)?
    1. Why are people placed at your site versus other sites?
    2. How is availability determined at your site?
    3. When are people dismissed or asked to leave your site? Is there a time limit, behavior, or situation that would prompt their departure from the site?
11. What makes your HRH program different from other housing options for people who are unstably housed?
12. How has drug use changed among residents since they have been placed in your HRH program?
    1. Probe: Type of drug, increase, decrease, risk?
    2. How do you handle emergencies on site?
    3. How do you handle violence on site?
13. If one, of your residents wanted to start MOUD, how would they go about that process?
    1. What types of MOUD? (ask about differences with bupe, methadone and vivitrol )
    2. Can they do this off site or on site?
    3. How do residents access medications? Delivery? Pharmacies?
14. Tell me about the changes that you have observed with respect to MOUD since people have re-located to your HRH location?
15. Have you noticed any other changes for residents since they have been placed in your HRH location?
16. How do you feel the needs of people living in your HRH location have changed over time?
17. Are needs different at day vs night?
    1. How have people formed “community” at your HRH location, what does that look like?
18. What do you need to meet the evolving needs of your residents?
    1. What is currently in existence to meet the needs of your residents
    2. What suggestions do you have to better meet the needs of your residents?
19. Is there anything else that you’d like to share today?
